# Supplementary material for: A genomic amplification affecting a carboxylesterase gene cluster confers organophosphate resistance in the mosquito Aedes aegypti: From genomic characterization to high‐throughput field detection
Source: Evol Appl. 2021 Feb 16;14(4):1009–22. doi: 10.1111/eva.13177 (PMC8061265; doi:10.1111/eva.13177)
Supplement: Supplementary file 1 — Fig S1 [file EVA-14-1009-s004.pdf]

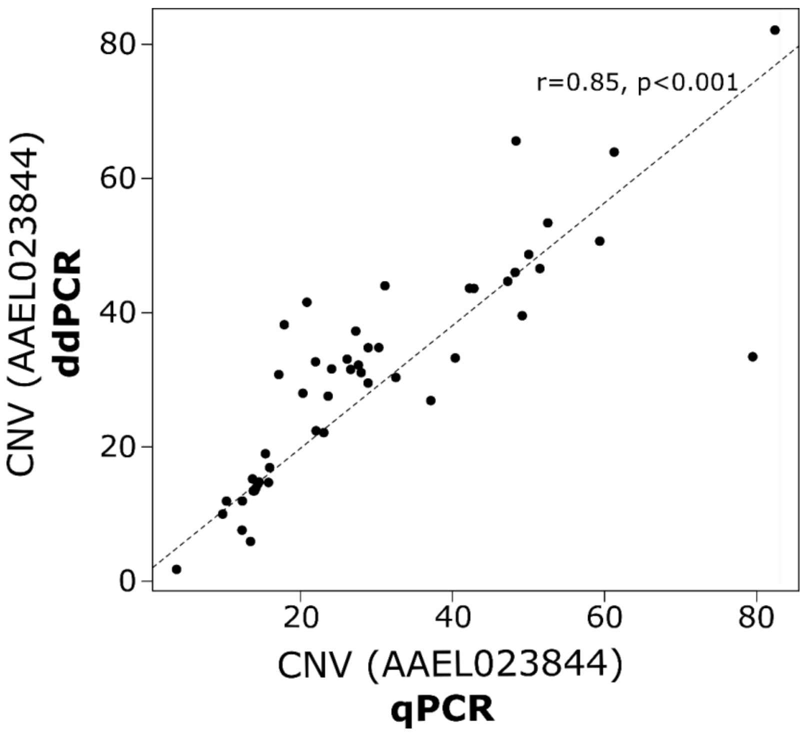

FIGURE S1. Correlation between CNV obtained from digital droplet qPCR and standard Sybergreen qPCR for the gene CCEAE3A (AAEL023844). CNV are expressed as normalized gDNA quantity relative to the fully susceptible line Bora-Bora. Each dot stands for a single positive mosquito.
