# Supplementary material for: A genomic amplification affecting a carboxylesterase gene cluster confers organophosphate resistance in the mosquito Aedes aegypti: From genomic characterization to high‐throughput field detection
Source: Evol Appl. 2021 Feb 16;14(4):1009–22. doi: 10.1111/eva.13177 (PMC8061265; doi:10.1111/eva.13177)
Supplement: Supplementary file 2 — Table S1 [file EVA-14-1009-s003.docx]

**Table S1**. Localization and collection date of populations used for building the Laos Composite Population (LCP) and for studying the prevalence of amplified carboxylcholinesterase genes (CCEs) in South- East Asia. Each population used to build the LCP was composed of individuals sampled from 2 to more than 10 breeding sites within a 100 m distance approximately.

|  | **Country** | **Province** | **District** | **Village** | **Collection date** | **GPS Latitude** | **GPS Longitude** |
| --- | --- | --- | --- | --- | --- | --- | --- |
| **Populations collected to  build the LCP** | LAOS | Xayaboury | Borten | Taling | juil-14 | 17,784729 | 101,170521 |
|  |  | Luang Prabang | Luang Prabang | Khomkhuang | juil-14 | 19,902775 | 102,156213 |
|  |  | Luang Prabang | Luang Prabang | Thatnoy | juil-14 | 19,531432 | 102,075364 |
|  |  | Luang Prabang | Luang Prabang | Thongchaleun | juil-14 | 19,887366 | 102,132352 |
|  |  | Vientiane Capital | Xaithany | Oudomphon | août-14 | 18,125733 | 102,665011 |
|  |  | Vientiane Capital | Xaithany | Phailom | août-14 | 18,057037 | 102,774993 |
|  |  | Vientiane Capital | Chanthabouly | Dongpalab | août-14 | 17,988083 | 102,605268 |
|  |  | Vientiane Capital | Sisattanak | Kao-gnot | août-14 | 17,962684 | 102,615035 |
|  |  | Saravane | Lakhonepheng | Lakhonepheng | sept-14 | 15,485507 | 105,403469 |
|  |  | Saravane | Vapi | Khonsaiy | sept-14 | 15,414079 | 105,541816 |
|  |  | Attapeu | Samakheexay | Xaysa-art | sept-14 | 14,484109 | 106,501415 |
| **Natural populations collected to study the prevalence of CCEs amplifications** | LAOS | Xayaboury (XA) | Xayaboury | Xayaboury | mai-17 | 19.259737 | 101.700310 |
|  |  | Bokeo (BO) | Huayxay | Huayxay | mai-17 | 20.46610 | 100.451431 |
|  |  | Luang Namtha (LU) | Luang Namtha | Luang Namtha | mai-17 | 20.896901 | 101.043613 |
|  |  | Khammouane (KH) | Mahaxay | Mahaxay | juin-17 | 17.408145 | 105.199757 |
|  |  | Vientiane Province (VP) | Vangvieng | Viengkeo | juin-17 | 18.955764 | 102.441823 |
|  |  | Vientiane Capital (VC) | Sisattanak | Saphanthong Tai | juil-17 | 17.936975 | 102.620024 |
|  |  | Champasack (CH) | Pakse | Pakse | juil-17 | 15.119974 | 105.788383 |
|  | THAILAND | Patthalung (PA) | / | / | juil-14 | 7.404995 | 100.222014 |
|  |  | Phetchaburi (PH) | / | / | juil-14 | 12.893522 | 99.866113 |
|  |  | Nakhon Sawan (NA) | / | / | juil-14 | 15.696300 | 100,173759 |
|  |  | Roi Et (RO) | / | / | juil-17 | 15.977956 | 103.816657 |
|  |  | Khon Kaen (KH) | / | / | juil-17 | 16.515712 | 102.642898 |
|  | CAMBODIA | Phnom Penh (PHN) | Doun Penh | Srah Chak | juil-17 | 11.561063 | 104.932126 |
|  |  | Kampong Cham (KA) | Kampong Cham | Boeng Kok | juil-17 | 11.979407 | 105.443000 |
