# Supplementary material for: A genomic amplification affecting a carboxylesterase gene cluster confers organophosphate resistance in the mosquito Aedes aegypti: From genomic characterization to high‐throughput field detection
Source: Evol Appl. 2021 Feb 16;14(4):1009–22. doi: 10.1111/eva.13177 (PMC8061265; doi:10.1111/eva.13177)
Supplement: Supplementary file 3 — Table S2 [file EVA-14-1009-s002.docx]

**Table S2**. Primers and probes used in this study

| **Methods** | | **Gene** | **Tm (°C)** | **Forward (5'-3')** | **Reverse (5'-3')** | **Product  length (bp)** |
| --- | --- | --- | --- | --- | --- | --- |
| **Quantitative PCR** | | AAEL005950* | 60 | TCCGGTTCCGTCTGGTATCT | GTGTGTGTAACGGCTCCAGA | 186 |
|  |  | AAEL023844* | 54 | TCTAAGAAACCCGAATATGACG | TTGAGGAGGCACGAACAG | 130 |
|  |  | CYP4D39** | 60 | AGTCCTGGAAGTTCTGCACG | AAGGCGACTTTCCGACGAAT | 132 |
|  |  | AAEL019678** | 60 | TTTGGCGATCGGTCTACAGG | GGTGAAACTCAATGCGATTCTT | 180 |
|  |  | CCEAE1A ** | 58 | TGAATGAAAGCGTGGGTGGT | TGCTTGTGAGTACTGTCTGACT | 182 |
| **Taqman assay** | **primers** | CYP4D39** | 60 | AGTCCTGGAAGTTCTGCACG | AAGGCGACTTTCCGACGAAT | 132 |
|  |  | AAEL023844** | 60 | TATAGCAGGAAGCGGCGATG | AATCCCAAGGGACCCAATCG | 102 |
|  | **probes** | CYP4D39** | 60 | [HEX]AAGGAGGCAAACCCCGATAA[BHQ1] |  |  |
|  |  | AAEL023844** | 60 | [FAM]TATAGTGCAGGAGGGGGTCA[BHQ1] |  |  |

*Faucon et al., 2015

**Designed in this study

Faucon, F., Dusfour, I., Gaude, T., Navratil, V., Boyer, F., Chandre, F., … David, J. (2015). Unravelling genomic changes associated with insecticide resistance in the dengue mosquito Aedes aegypti by deep targeted sequencing. *Genome Research*, (August), 1347–1359. doi: 10.1101/gr.189225.115
