# Supplementary material for: A genomic amplification affecting a carboxylesterase gene cluster confers organophosphate resistance in the mosquito Aedes aegypti: From genomic characterization to high‐throughput field detection
Source: Evol Appl. 2021 Feb 16;14(4):1009–22. doi: 10.1111/eva.13177 (PMC8061265; doi:10.1111/eva.13177)
Supplement: Supplementary file 4 — Table S3 [file EVA-14-1009-s005.docx]

| **Gene ID** | **Chr** | **Start** | **End** | **Strand** | **Description** | **Gene name** |  | **G6-Mala Vs Bora-Bora** | | |  | **G6-Mala Vs G6-NS** | | |  | **associated resistance mechanism** |
| --- | --- | --- | --- | --- | --- | --- | --- | --- | --- | --- | --- | --- | --- | --- | --- | --- |
|  |  |  |  |  |  |  |  | **P value (adjusted)** | **Fold Change** | **Log_2_ Fold Change** |  | **P value (adjusted)** | **Fold Change** | **Log_2_ Fold Change** |  |  |
| AAEL017987 | 1 | 13145939 | 13159171 | - |  |  |  | 1,50E-04 | -9,20 | -3,20 |  | 1,50E-04 | -13,55 | -3,76 |  |  |
| AAEL020941 | 1 | 18921560 | 19079417 | - |  |  |  | 1,50E-04 | -5,38 | -2,43 |  | 1,50E-04 | -5,79 | -2,53 |  |  |
| AAEL027257 | 1 | 21508471 | 21530418 | - |  |  |  | 1,50E-04 | 4,49 | 2,17 |  | 1,50E-04 | 3,78 | 1,92 |  |  |
| AAEL013262 | 1 | 32344860 | 32345614 | - |  |  |  | 1,50E-04 | 6,48 | 2,70 |  | 1,50E-04 | 10,27 | 3,36 |  |  |
| AAEL021791 | 1 | 34810919 | 34831349 | - |  |  |  | 1,53E-04 | -8,46 | -3,08 |  | 1,50E-04 | -10,09 | -3,34 |  |  |
| AAEL001319 | 1 | 34932984 | 34946537 | - |  |  |  | 1,50E-04 | -3,90 | -1,96 |  | 1,50E-04 | -4,40 | -2,14 |  |  |
| AAEL020641 | 1 | 37420448 | 37421223 | - |  |  |  | 2,28E-04 | 4,77 | 2,25 |  | 5,04E-04 | 3,96 | 1,99 |  |  |
| **AAEL006818** | **1** | **53127847** | **53152580** | **+** | **glutathione S-transferase, putative** |  |  | **1,50E-04** | **56,65** | **5,82** |  | **1,50E-04** | **13,26** | **3,73** |  | **detoxification** |
| AAEL025784 | 1 | 72769112 | 73024741 | + |  |  |  | 1,54E-04 | -4,80 | -2,26 |  | 1,50E-04 | -5,55 | -2,47 |  |  |
| AAEL020748 | 1 | 73082036 | 73158148 | + |  |  |  | 1,95E-04 | -4,36 | -2,12 |  | 1,50E-04 | -6,99 | -2,81 |  |  |
| AAEL005431 | 1 | 78808137 | 78809601 | - | Clip-Domain Serine Protease family B. | CLIPB37 |  | 1,50E-04 | 4,10 | 2,04 |  | 1,50E-04 | 3,46 | 1,79 |  |  |
| AAEL021785 | 1 | 84127035 | 84277965 | + |  |  |  | 1,62E-04 | -3,12 | -1,64 |  | 1,50E-04 | -6,21 | -2,64 |  |  |
| AAEL023438 | 1 | 84298555 | 84321535 | + |  |  |  | 2,88E-04 | -3,82 | -1,94 |  | 1,97E-04 | -4,29 | -2,10 |  |  |
| AAEL009484 | 1 | 98328899 | 98345264 | - |  |  |  | 3,99E-04 | -7,53 | -2,91 |  | 1,50E-04 | -23,64 | -4,56 |  |  |
| AAEL006376 | 1 | 101053170 | 101054242 | - | trypsin, putative |  |  | 1,56E-04 | 3,55 | 1,83 |  | 1,50E-04 | 5,83 | 2,54 |  |  |
| AAEL004240 | 1 | 104236836 | 104238981 | - | gamma glutamyl transpeptidases |  |  | 1,50E-04 | -10,39 | -3,38 |  | 1,64E-04 | -5,65 | -2,50 |  |  |
| AAEL026432 | 1 | 105525675 | 105584949 | - |  |  |  | 1,50E-04 | -3,86 | -1,95 |  | 1,50E-04 | -6,07 | -2,60 |  |  |
| AAEL011586 | 1 | 119918678 | 120285270 | + |  |  |  | 2,18E-04 | -3,11 | -1,64 |  | 2,31E-04 | -3,06 | -1,61 |  |  |
| AAEL006168 | 1 | 126386712 | 126388196 | + | Clip-Domain Serine Protease family B. | CLIPB42 |  | 1,50E-04 | 3,54 | 1,82 |  | 1,50E-04 | 4,82 | 2,27 |  |  |
| AAEL020007 | 1 | 129269074 | 129302734 | + |  |  |  | 4,11E-04 | -3,67 | -1,88 |  | 2,22E-04 | -4,23 | -2,08 |  |  |
| AAEL003049 | 1 | 130920943 | 130922491 | + | pupal cuticle protein 78E, putative |  |  | 1,56E-04 | -6,52 | -2,71 |  | 1,50E-04 | -13,03 | -3,70 |  | cuticle change |
| AAEL003041 | 1 | 131372437 | 131400400 | + |  |  |  | 1,50E-04 | -5,22 | -2,38 |  | 1,50E-04 | -8,34 | -3,06 |  |  |
| AAEL022352 | 1 | 131450673 | 131497719 | + |  |  |  | 1,50E-04 | -3,55 | -1,83 |  | 1,50E-04 | -6,86 | -2,78 |  |  |
| AAEL012245 | 1 | 146979768 | 147016446 | + |  |  |  | 1,50E-04 | -3,82 | -1,93 |  | 1,50E-04 | -4,98 | -2,32 |  |  |
| AAEL009580 | 1 | 147187735 | 147189357 | + |  |  |  | 1,50E-04 | -10,25 | -3,36 |  | 1,50E-04 | -21,32 | -4,41 |  |  |
| AAEL014820 | 1 | 149177812 | 149226500 | + |  |  |  | 1,50E-04 | -3,27 | -1,71 |  | 1,50E-04 | -3,72 | -1,89 |  |  |
| AAEL014823 | 1 | 149348820 | 149402818 | - |  |  |  | 1,50E-04 | -4,92 | -2,30 |  | 1,50E-04 | -7,20 | -2,85 |  |  |
| AAEL013766 | 1 | 162616995 | 162692189 | - |  |  |  | 1,50E-04 | -5,74 | -2,52 |  | 1,50E-04 | -11,65 | -3,54 |  |  |
| AAEL008414 | 1 | 173712125 | 173902662 | - |  |  |  | 1,51E-04 | -14,80 | -3,89 |  | 4,43E-04 | -6,16 | -2,62 |  |  |
| AAEL003632 | 1 | 188950538 | 188956330 | + | Clip-Domain Serine Protease family B. | CLIPB39 |  | 1,50E-04 | 5,99 | 2,58 |  | 1,50E-04 | 9,49 | 3,25 |  |  |
| AAEL015465 | 1 | 188978005 | 188989323 | + | clip-domain serine protease, putative |  |  | 1,50E-04 | 3,88 | 1,96 |  | 1,50E-04 | 3,50 | 1,81 |  |  |
| AAEL017533 | 1 | 195104864 | 195106843 | + |  |  |  | 1,73E-04 | -6,49 | -2,70 |  | 1,50E-04 | -11,33 | -3,50 |  |  |
| AAEL005792 | 1 | 201595574 | 201613893 | + | Clip-Domain Serine Protease family E. Protease homologue. | CLIPE8 |  | 1,50E-04 | 17,91 | 4,16 |  | 1,50E-04 | 7,84 | 2,97 |  |  |
| AAEL025806 | 1 | 205184850 | 205188012 | + |  |  |  | 1,50E-04 | -6,62 | -2,73 |  | 1,50E-04 | -9,58 | -3,26 |  |  |
| AAEL014945 | 1 | 213611425 | 213616543 | + |  |  |  | 1,50E-04 | 3,79 | 1,92 |  | 1,50E-04 | 3,72 | 1,89 |  |  |
| AAEL007483 | 1 | 216289250 | 216324311 | + |  |  |  | 1,50E-04 | -10,47 | -3,39 |  | 1,50E-04 | -12,30 | -3,62 |  |  |
| AAEL002972 | 1 | 261341183 | 261351659 | + | brain chitinase and chia |  |  | 1,56E-04 | -4,72 | -2,24 |  | 1,50E-04 | -10,81 | -3,43 |  |  |
| AAEL014893 | 1 | 271328495 | 271330383 | - | cytochrome P450 | CYP6BB2 |  | 1,50E-04 | -3,12 | -1,64 |  | 1,50E-04 | -3,35 | -1,75 |  | detoxification |
| AAEL015163 | 1 | 276011236 | 276012315 | - | cuticle protein, putative |  |  | 5,41E-04 | -4,14 | -2,05 |  | 1,84E-04 | -5,65 | -2,50 |  | cuticle change |
| AAEL009784 | 1 | 276109259 | 276110249 | - | cuticle protein, putative |  |  | 7,53E-04 | 5,27 | 2,40 |  | 1,59E-04 | 9,91 | 3,31 |  |  |
| AAEL007992 | 1 | 283123380 | 283139443 | + | trypsin, putative |  |  | 1,50E-04 | 3,73 | 1,90 |  | 1,50E-04 | 3,36 | 1,75 |  |  |
| AAEL003262 | 1 | 291039926 | 291135913 | + | leucine-rich transmembrane protein |  |  | 1,56E-04 | -3,56 | -1,83 |  | 1,50E-04 | -5,79 | -2,53 |  |  |
| AAEL025140 | 1 | 305999815 | 306066595 | - |  |  |  | 1,50E-04 | -5,15 | -2,37 |  | 1,50E-04 | -6,38 | -2,67 |  |  |
| AAEL008473 | 2 | 19337484 | 19339403 | + | cysteine-rich venom protein, putative |  |  | 1,50E-04 | 12,89 | 3,69 |  | 1,50E-04 | 8,62 | 3,11 |  |  |
| AAEL025803 | 2 | 23759210 | 23760265 | + |  |  |  | 1,50E-04 | 93,83 | 6,55 |  | 2,69E-04 | 11,96 | 3,58 |  |  |
| AAEL006888 | 2 | 25520567 | 25605738 | + |  |  |  | 1,62E-04 | -3,62 | -1,86 |  | 1,50E-04 | -5,05 | -2,34 |  |  |
| AAEL017132 | 2 | 35588182 | 35590420 | - | C-Type Lysozyme (Lys-C). | LYSC4 |  | 1,50E-04 | 3,50 | 1,81 |  | 1,50E-04 | 4,51 | 2,17 |  |  |
| AAEL011449 | 2 | 37031615 | 37057848 | - |  |  |  | 1,50E-04 | -4,36 | -2,12 |  | 1,50E-04 | -7,33 | -2,87 |  |  |
| AAEL011444 | 2 | 37431013 | 37432379 | - | pupal cuticle protein, putative |  |  | 3,88E-04 | -7,31 | -2,87 |  | 5,44E-04 | -6,69 | -2,74 |  | cuticle change |
| AAEL013515 | 2 | 37553275 | 37553889 | - | pupal cuticle protein, putative |  |  | 1,50E-04 | -3,76 | -1,91 |  | 1,50E-04 | -3,33 | -1,74 |  | cuticle change |
| AAEL001745 | 2 | 37872708 | 37888083 | + | candidate tumor suppressor protein |  |  | 1,50E-04 | -5,60 | -2,48 |  | 1,50E-04 | -8,97 | -3,17 |  |  |
| AAEL001437 | 2 | 47004237 | 47644489 | + |  |  |  | 1,50E-04 | -5,26 | -2,40 |  | 1,50E-04 | -8,48 | -3,08 |  |  |
| AAEL001396 | 2 | 47801236 | 47847673 | + | mandelate racemase |  |  | 1,50E-04 | -3,07 | -1,62 |  | 1,50E-04 | -4,59 | -2,20 |  |  |
| AAEL013556 | 2 | 58002198 | 58004004 | - | cytochrome P450 | CYP4J15 |  | 1,58E-04 | -8,92 | -3,16 |  | 1,50E-04 | -24,49 | -4,61 |  | detoxification |
| AAEL008285 | 2 | 67420433 | 67428123 | - | pupal cuticle protein, putative |  |  | 1,50E-04 | -4,38 | -2,13 |  | 1,50E-04 | -8,19 | -3,03 |  | cuticle change |
| AAEL004897 | 2 | 71090279 | 71297267 | + | brain chitinase and chia |  |  | 1,50E-04 | -3,15 | -1,66 |  | 1,50E-04 | -4,50 | -2,17 |  |  |
| AAEL028005 | 2 | 75979586 | 75990992 | - |  |  |  | 1,50E-04 | 4,22 | 2,08 |  | 1,50E-04 | 4,35 | 2,12 |  |  |
| AAEL018241 | 2 | 76262424 | 76296157 | - |  |  |  | 1,50E-04 | 7,30 | 2,87 |  | 1,54E-04 | 4,52 | 2,18 |  |  |
| AAEL002467 | 2 | 78797991 | 78799643 | + |  |  |  | 1,50E-04 | 47,90 | 5,58 |  | 2,20E-04 | 11,12 | 3,48 |  |  |
| AAEL011504 | 2 | 81793985 | 81796195 | - | pupal cuticle protein, putative |  |  | 1,84E-04 | -3,35 | -1,75 |  | 1,50E-04 | -15,22 | -3,93 |  | cuticle change |
| AAEL001677 | 2 | 85966047 | 85966856 | - |  |  |  | 1,50E-04 | -4,78 | -2,26 |  | 1,50E-04 | -9,37 | -3,23 |  |  |
| AAEL001683 | 2 | 86005275 | 86006231 | + |  |  |  | 1,50E-04 | -4,87 | -2,28 |  | 1,50E-04 | -10,67 | -3,42 |  |  |
| AAEL027545 | 2 | 96105283 | 96132137 | + |  |  |  | 1,52E-04 | -3,04 | -1,60 |  | 1,50E-04 | -4,46 | -2,16 |  |  |
| AAEL013853 | 2 | 100166111 | 100200638 | + | C-Type Lectin (CTL) - galactose binding. | CTLGA2 |  | 1,52E-04 | -3,50 | -1,81 |  | 1,50E-04 | -5,05 | -2,34 |  |  |
| AAEL004249 | 2 | 118669190 | 118687796 | - |  |  |  | 1,52E-04 | -3,50 | -1,81 |  | 1,50E-04 | -6,26 | -2,65 |  |  |
| AAEL025272 | 2 | 118885668 | 118887004 | - |  |  |  | 1,50E-04 | 4,05 | 2,02 |  | 1,50E-04 | 4,45 | 2,15 |  |  |
| AAEL021510 | 2 | 126800443 | 126902154 | - |  |  |  | 1,50E-04 | -4,09 | -2,03 |  | 1,50E-04 | -4,80 | -2,26 |  |  |
| AAEL013303 | 2 | 130250613 | 130253083 | + |  |  |  | 1,50E-04 | -4,27 | -2,09 |  | 1,50E-04 | -7,83 | -2,97 |  |  |
| AAEL014078 | 2 | 132319515 | 132330329 | + | serine protease inhibitor, serpin |  |  | 1,50E-04 | 3,85 | 1,94 |  | 1,50E-04 | 3,89 | 1,96 |  |  |
| AAEL016992 | 2 | 133740815 | 134170366 | + |  |  |  | 1,50E-04 | -6,40 | -2,68 |  | 1,50E-04 | -9,17 | -3,20 |  |  |
| AAEL025092 | 2 | 155948131 | 155949142 | - |  |  |  | 1,50E-04 | -6,38 | -2,67 |  | 1,50E-04 | -10,16 | -3,35 |  |  |
| AAEL010125 | 2 | 161626755 | 161628833 | - | leucine-rich immune protein (Coil-less) | LRIM17 |  | 1,50E-04 | 3,45 | 1,79 |  | 1,50E-04 | 3,17 | 1,67 |  |  |
| AAEL010134 | 2 | 162088434 | 162089152 | + | Pupal cuticle protein, putative |  |  | 1,60E-04 | -4,94 | -2,30 |  | 1,50E-04 | -12,73 | -3,67 |  | cuticle change |
| AAEL015019 | 2 | 165237023 | 165240275 | + |  |  |  | 1,51E-04 | -3,39 | -1,76 |  | 1,50E-04 | -3,55 | -1,83 |  |  |
| AAEL011766 | 2 | 168708951 | 168764477 | + |  |  |  | 1,50E-04 | -3,49 | -1,80 |  | 1,50E-04 | -4,81 | -2,27 |  |  |
| AAEL011771 | 2 | 168881061 | 168885427 | + |  |  |  | 1,50E-04 | -3,69 | -1,88 |  | 1,50E-04 | -5,16 | -2,37 |  |  |
| **AAEL015304** | **2** | **174336232** | **174346094** | **-** | **CCE-Like (manual annotation)** |  |  | **1,91E-04** | **3,77** | **1,92** |  | **3,85E-04** | **3,18** | **1,67** |  | **detoxification** |
| **AAEL019679** | **2** | **174358955** | **174365657** | **+** | **carboxycholinesterase** |  |  | **1,50E-04** | **4,68** | **2,23** |  | **1,50E-04** | **3,35** | **1,75** |  | **detoxification** |
| **AAEL019678** | **2** | **174359042** | **174377560** | **+** | **carboxycholinesterase** |  |  | **1,50E-04** | **5,62** | **2,49** |  | **1,50E-04** | **5,19** | **2,38** |  | **detoxification** |
| **AAEL005123** | **2** | **174365599** | **174375354** | **-** | **CCE-Like (manual annotation)** |  |  | **1,50E-04** | **8,27** | **3,05** |  | **1,50E-04** | **6,31** | **2,66** |  | **detoxification** |
| **AAEL023844** | **2** | **174400171** | **174416020** | **-** | **carboxycholinesterase** |  |  | **1,50E-04** | **8,84** | **3,14** |  | **1,50E-04** | **10,84** | **3,44** |  | **detoxification** |
| AAEL002360 | 2 | 175126538 | 175127614 | + | serine-type enodpeptidase, |  |  | 1,53E-04 | -6,74 | -2,75 |  | 1,50E-04 | -10,98 | -3,46 |  |  |
| AAEL022845 | 2 | 177534220 | 177582164 | + |  |  |  | 5,17E-04 | -4,43 | -2,15 |  | 1,51E-04 | -9,05 | -3,18 |  |  |
| AAEL021173 | 2 | 180153963 | 180168571 | - |  |  |  | 1,50E-04 | 6,20 | 2,63 |  | 1,50E-04 | 4,86 | 2,28 |  |  |
| AAEL021609 | 2 | 180168609 | 180177927 | - |  |  |  | 1,50E-04 | 6,49 | 2,70 |  | 1,50E-04 | 4,72 | 2,24 |  |  |
| AAEL011458 | 2 | 187028726 | 187030014 | - |  |  |  | 5,98E-04 | -4,61 | -2,21 |  | 1,52E-04 | -9,15 | -3,19 |  |  |
| AAEL013511 | 2 | 189776774 | 189777679 | + |  |  |  | 1,50E-04 | -6,20 | -2,63 |  | 1,50E-04 | -13,28 | -3,73 |  |  |
| AAEL013217 | 2 | 199640033 | 199652647 | + | odorant receptor (Or31) | GPROR32 |  | 2,21E-04 | 6,54 | 2,71 |  | 1,56E-04 | 9,24 | 3,21 |  |  |
| AAEL001392 | 2 | 205927148 | 205928997 | + |  |  |  | 1,53E-04 | 12,76 | 3,67 |  | 3,27E-04 | 6,83 | 2,77 |  |  |
| AAEL011440 | 2 | 225608336 | 225638207 | + |  |  |  | 1,52E-04 | -4,70 | -2,23 |  | 1,50E-04 | -7,39 | -2,88 |  |  |
| AAEL008066 | 2 | 230211060 | 230376863 | + | trkB protein, putative |  |  | 1,50E-04 | -4,55 | -2,19 |  | 1,50E-04 | -5,48 | -2,45 |  |  |
| AAEL008069 | 2 | 230912634 | 230954150 | + | notch |  |  | 1,52E-04 | -3,79 | -1,92 |  | 1,50E-04 | -4,77 | -2,25 |  |  |
| AAEL014349 | 2 | 238627219 | 238629106 | + | Clip-Domain Serine Protease family B. | CLIPB15 |  | 1,50E-04 | 3,25 | 1,70 |  | 1,50E-04 | 3,06 | 1,61 |  |  |
| AAEL002654 | 2 | 245638274 | 245649761 | + |  |  |  | 1,84E-04 | -8,77 | -3,13 |  | 1,52E-04 | -12,88 | -3,69 |  |  |
| AAEL010139 | 2 | 264662071 | 264678075 | + | serine protease, putative |  |  | 4,38E-04 | 6,59 | 2,72 |  | 1,53E-04 | 13,68 | 3,77 |  |  |
| AAEL010137 | 2 | 265243282 | 265244106 | - | ketoreductase, putative |  |  | 1,50E-04 | -16,29 | -4,03 |  | 1,58E-04 | -9,17 | -3,20 |  |  |
| AAEL011375 | 2 | 266223135 | 266226443 | + | trypsin, putative |  |  | 1,74E-04 | -5,51 | -2,46 |  | 1,62E-04 | -5,94 | -2,57 |  |  |
| AAEL004292 | 2 | 293325027 | 293333699 | + |  |  |  | 1,50E-04 | -10,80 | -3,43 |  | 1,50E-04 | -26,21 | -4,71 |  |  |
| AAEL002406 | 2 | 303072615 | 303074045 | + |  |  |  | 1,50E-04 | 10,97 | 3,46 |  | 1,50E-04 | 13,60 | 3,77 |  |  |
| AAEL017023 | 2 | 309020232 | 309042731 | + |  |  |  | 1,50E-04 | 3,56 | 1,83 |  | 1,50E-04 | 3,50 | 1,81 |  |  |
| AAEL013222 | 2 | 314391509 | 314416287 | - |  |  |  | 1,50E-04 | -4,03 | -2,01 |  | 1,50E-04 | -8,02 | -3,00 |  |  |
| AAEL013367 | 2 | 323288657 | 323896767 | + |  |  |  | 1,85E-04 | -4,75 | -2,25 |  | 1,51E-04 | -6,87 | -2,78 |  |  |
| AAEL014754 | 2 | 363138980 | 363149168 | - |  |  |  | 1,50E-04 | 6,04 | 2,59 |  | 1,50E-04 | 5,84 | 2,55 |  |  |
| AAEL003272 | 2 | 366354773 | 366369822 | + | pupal cuticle protein 78E, putative |  |  | 2,89E-04 | -8,46 | -3,08 |  | 1,57E-04 | -13,88 | -3,80 |  | cuticle change |
| AAEL003259 | 2 | 366562297 | 366570172 | + | pupal cuticle protein 78E, putative |  |  | 1,51E-04 | -6,06 | -2,60 |  | 1,50E-04 | -9,90 | -3,31 |  | cuticle change |
| AAEL001233 | 2 | 372264358 | 372292559 | - | Clip-Domain Serine Protease family E. Protease homologue. | CLIPE9 |  | 1,50E-04 | -3,18 | -1,67 |  | 1,50E-04 | -4,79 | -2,26 |  |  |
| AAEL001822 | 2 | 374871931 | 374873949 | + | glucosyl/glucuronosyl transferases |  |  | 1,56E-04 | -10,67 | -3,42 |  | 1,67E-04 | -9,11 | -3,19 |  | detoxification |
| AAEL001836 | 2 | 375509589 | 375510577 | + | odorant-binding protein 56a, putative |  |  | 1,59E-04 | -7,16 | -2,84 |  | 1,52E-04 | -8,34 | -3,06 |  |  |
| **AAEL010158** | **2** | **380784709** | **380786544** | **-** | **cytochrome P450** | **CYP6N17** |  | **1,59E-04** | **8,64** | **3,11** |  | **1,60E-04** | **8,49** | **3,09** |  | **detoxification** |
| AAEL012268 | 2 | 384387914 | 384414521 | - | brain chitinase and chia |  |  | 8,35E-04 | -3,16 | -1,66 |  | 1,52E-04 | -5,77 | -2,53 |  |  |
| AAEL025155 | 2 | 386731262 | 386733266 | + |  |  |  | 1,54E-04 | -6,95 | -2,80 |  | 1,50E-04 | -8,52 | -3,09 |  |  |
| AAEL006971 | 2 | 398736089 | 398737369 | + |  |  |  | 1,50E-04 | 15,50 | 3,95 |  | 1,50E-04 | 26,65 | 4,74 |  |  |
| AAEL006969 | 2 | 398758059 | 398762255 | - |  |  |  | 1,50E-04 | 6,46 | 2,69 |  | 1,50E-04 | 3,78 | 1,92 |  |  |
| AAEL020192 | 2 | 407754294 | 407755867 | - |  |  |  | 1,50E-04 | 6,36 | 2,67 |  | 1,50E-04 | 5,59 | 2,48 |  |  |
| AAEL010682 | 2 | 418269136 | 418275507 | - | armc4 |  |  | 1,50E-04 | 4,89 | 2,29 |  | 1,93E-04 | 3,42 | 1,77 |  |  |
| AAEL009131 | 2 | 418865260 | 418867369 | - | cytochrome P450 | CYP6Z8 |  | 1,52E-04 | -3,77 | -1,92 |  | 1,55E-04 | -3,48 | -1,80 |  | detoxification |
| AAEL024828 | 2 | 428843770 | 428848867 | - |  |  |  | 1,55E-04 | -3,53 | -1,82 |  | 1,50E-04 | -7,76 | -2,96 |  |  |
| AAEL017402 | 2 | 428865978 | 428867094 | + |  |  |  | 1,50E-04 | -4,38 | -2,13 |  | 1,50E-04 | -9,06 | -3,18 |  |  |
| AAEL025608 | 2 | 428882059 | 428882784 | + | Putative cuticle protein |  |  | 1,51E-04 | -3,00 | -1,59 |  | 1,50E-04 | -4,16 | -2,06 |  | cuticle change |
| AAEL020593 | 2 | 428930713 | 428931869 | + |  |  |  | 1,50E-04 | -3,32 | -1,73 |  | 1,50E-04 | -3,69 | -1,88 |  |  |
| AAEL002231 | 2 | 429170789 | 429171449 | + | cuticle protein, putative |  |  | 1,52E-04 | -5,42 | -2,44 |  | 3,61E-04 | -3,41 | -1,77 |  | cuticle change |
| AAEL021995 | 2 | 432120495 | 432132460 | + |  |  |  | 1,52E-04 | -3,46 | -1,79 |  | 1,50E-04 | -4,75 | -2,25 |  |  |
| AAEL027439 | 2 | 432348471 | 432350650 | + |  |  |  | 1,59E-04 | -4,66 | -2,22 |  | 1,50E-04 | -7,72 | -2,95 |  |  |
| AAEL002655 | 2 | 433717688 | 433719537 | - | matrix metalloproteinase |  |  | 1,50E-04 | 7,29 | 2,87 |  | 1,50E-04 | 8,73 | 3,13 |  |  |
| AAEL005748 | 2 | 436023103 | 436038301 | - | elastase, putative |  |  | 1,50E-04 | 4,60 | 2,20 |  | 1,50E-04 | 5,96 | 2,58 |  |  |
| AAEL006533 | 2 | 468786124 | 468834837 | - | Ets domain-containing protein |  |  | 2,15E-04 | 5,86 | 2,55 |  | 4,39E-04 | 4,79 | 2,26 |  |  |
| AAEL009384 | 3 | 8158773 | 8160684 | + | fibrinogen and fibronectin |  |  | 1,50E-04 | 338,37 | 8,40 |  | 1,50E-04 | 60,46 | 5,92 |  |  |
| AAEL027985 | 3 | 10074481 | 10076158 | + |  |  |  | 1,57E-04 | -3,71 | -1,89 |  | 2,32E-04 | -3,03 | -1,60 |  |  |
| AAEL010396 | 3 | 13370000 | 13380067 | - | secreted ferritin G subunit precursor, putative | |  | 1,50E-04 | 38,00 | 5,25 |  | 1,50E-04 | 38,13 | 5,25 |  |  |
| AAEL010393 | 3 | 13380903 | 13393919 | + | ferritin subunit, putative |  |  | 1,50E-04 | 20,03 | 4,32 |  | 1,50E-04 | 31,64 | 4,98 |  |  |
| AAEL010397 | 3 | 13744165 | 13774455 | + |  |  |  | 1,50E-04 | -3,13 | -1,65 |  | 1,50E-04 | -4,26 | -2,09 |  |  |
| AAEL017345 | 3 | 14728190 | 14729443 | + |  |  |  | 1,50E-04 | 4,41 | 2,14 |  | 1,50E-04 | 5,09 | 2,35 |  |  |
| AAEL008106 | 3 | 14729680 | 14730939 | + |  |  |  | 1,50E-04 | 4,23 | 2,08 |  | 1,50E-04 | 4,54 | 2,18 |  |  |
| AAEL017144 | 3 | 14775074 | 14776091 | + |  |  |  | 1,50E-04 | 3,45 | 1,79 |  | 1,50E-04 | 5,05 | 2,34 |  |  |
| AAEL025531 | 3 | 14782403 | 14782837 | - |  |  |  | 1,50E-04 | 18,11 | 4,18 |  | 1,50E-04 | 26,33 | 4,72 |  |  |
| AAEL021929 | 3 | 14793624 | 14794001 | + |  |  |  | 1,50E-04 | 14,87 | 3,89 |  | 1,50E-04 | 9,59 | 3,26 |  |  |
| AAEL017380 | 3 | 14834357 | 14834926 | - |  |  |  | 4,13E-04 | 12,52 | 3,65 |  | 2,06E-04 | 17,43 | 4,12 |  |  |
| AAEL023945 | 3 | 29108337 | 29109545 | - |  |  |  | 1,67E-04 | -9,78 | -3,29 |  | 1,69E-04 | -9,69 | -3,28 |  |  |
| AAEL023409 | 3 | 69993496 | 70005152 | - |  |  |  | 1,50E-04 | 55,85 | 5,80 |  | 2,82E-04 | 4,93 | 2,30 |  |  |
| AAEL025705 | 3 | 73720391 | 73722463 | + |  |  |  | 1,68E-04 | -4,27 | -2,09 |  | 1,50E-04 | -13,66 | -3,77 |  |  |
| AAEL014363 | 3 | 75230240 | 75232429 | + |  |  |  | 4,77E-04 | 3,07 | 1,62 |  | 2,25E-04 | 3,54 | 1,82 |  |  |
| AAEL005093 | 3 | 82887794 | 82897028 | - | Clip-Domain Serine Protease family B. | CLIPB46 |  | 1,50E-04 | 4,55 | 2,19 |  | 1,50E-04 | 4,58 | 2,19 |  |  |
| AAEL003888 | 3 | 84643785 | 84661591 | + | ubiquitin |  |  | 1,50E-04 | -3,78 | -1,92 |  | 1,50E-04 | -7,34 | -2,88 |  |  |
| AAEL004353 | 3 | 94406266 | 94407364 | - |  |  |  | 1,59E-04 | -3,20 | -1,68 |  | 1,50E-04 | -4,02 | -2,01 |  |  |
| AAEL000098 | 3 | 103726063 | 103830545 | + |  |  |  | 1,70E-04 | -7,99 | -3,00 |  | 1,50E-04 | -19,43 | -4,28 |  |  |
| AAEL022932 | 3 | 104646962 | 104797625 | + |  |  |  | 1,72E-04 | -3,73 | -1,90 |  | 1,52E-04 | -4,59 | -2,20 |  |  |
| AAEL014369 | 3 | 110873931 | 110908869 | + |  |  |  | 1,83E-04 | -8,63 | -3,11 |  | 1,50E-04 | -16,19 | -4,02 |  |  |
| AAEL000340 | 3 | 112007456 | 112025792 | + | cytochrome P450 |  |  | 1,50E-04 | -3,26 | -1,71 |  | 1,50E-04 | -3,19 | -1,67 |  | detoxification |
| AAEL004216 | 3 | 117407531 | 117426860 | + |  |  |  | 1,50E-04 | 21,91 | 4,45 |  | 1,50E-04 | 22,27 | 4,48 |  |  |
| AAEL002718 | 3 | 125588140 | 125711451 | + | chitin synthase |  |  | 1,50E-04 | -5,61 | -2,49 |  | 1,50E-04 | -8,34 | -3,06 |  | cuticle change |
| AAEL002780 | 3 | 129448530 | 129631424 | + |  |  |  | 1,75E-04 | -5,25 | -2,39 |  | 1,50E-04 | -13,35 | -3,74 |  |  |
| AAEL026093 | 3 | 135247319 | 135253549 | - |  |  |  | 1,50E-04 | -4,45 | -2,15 |  | 1,50E-04 | -9,81 | -3,29 |  |  |
| AAEL012082 | 3 | 160709199 | 160774878 | + | NF-180, putative |  |  | 1,50E-04 | -3,50 | -1,81 |  | 1,50E-04 | -5,93 | -2,57 |  |  |
| AAEL000496 | 3 | 163069876 | 163076838 | + |  |  |  | 2,26E-04 | 3,07 | 1,62 |  | 1,51E-04 | 4,44 | 2,15 |  |  |
| AAEL004003 | 3 | 166700020 | 166784334 | + | glucose dehydrogenase |  |  | 2,75E-04 | -3,59 | -1,84 |  | 1,50E-04 | -6,88 | -2,78 |  |  |
| AAEL006797 | 3 | 169999243 | 170218661 | + | F-box/leucine rich repeat protein |  |  | 1,78E-04 | -3,53 | -1,82 |  | 1,51E-04 | -4,55 | -2,19 |  |  |
| AAEL002092 | 3 | 177534227 | 177537162 | - | cuticle protein, putative |  |  | 1,50E-04 | -7,42 | -2,89 |  | 1,50E-04 | -9,13 | -3,19 |  | cuticle change |
| AAEL000428 | 3 | 186857871 | 186870670 | + | tryptophan 2,3-dioxygenase (TDO) | Tdo |  | 1,50E-04 | 3,12 | 1,64 |  | 1,50E-04 | 3,04 | 1,60 |  |  |
| AAEL007281 | 3 | 196223235 | 196299101 | + | stretchin-mlck |  |  | 1,50E-04 | -8,03 | -3,01 |  | 1,50E-04 | -12,77 | -3,67 |  |  |
| AAEL001951 | 3 | 227584945 | 227588623 | + | actin | Act-4 |  | 1,51E-04 | -11,73 | -3,55 |  | 1,50E-04 | -23,50 | -4,55 |  |  |
| AAEL005969 | 3 | 227937889 | 228001411 | - | phospholipase b, plb1 |  |  | 1,53E-04 | -4,01 | -2,00 |  | 1,50E-04 | -9,11 | -3,19 |  |  |
| AAEL011232 | 3 | 239508388 | 239525959 | - |  |  |  | 1,50E-04 | -4,43 | -2,15 |  | 1,50E-04 | -3,75 | -1,91 |  |  |
| AAEL008274 | 3 | 250453230 | 250466691 | + |  |  |  | 1,50E-04 | 3,49 | 1,80 |  | 1,50E-04 | 3,18 | 1,67 |  |  |
| AAEL007711 | 3 | 255486907 | 255488227 | + |  |  |  | 1,50E-04 | 6,07 | 2,60 |  | 1,50E-04 | 9,10 | 3,19 |  |  |
| AAEL002624 | 3 | 257262783 | 257264911 | - | serine protease |  |  | 1,50E-04 | 3,04 | 1,61 |  | 1,50E-04 | 3,08 | 1,62 |  |  |
| AAEL002585 | 3 | 257272883 | 257274316 | - | serine protease |  |  | 1,50E-04 | 4,14 | 2,05 |  | 1,50E-04 | 3,33 | 1,74 |  |  |
| AAEL002610 | 3 | 257284592 | 257286181 | - | serine protease |  |  | 1,50E-04 | 5,59 | 2,48 |  | 1,50E-04 | 5,44 | 2,44 |  |  |
| AAEL001510 | 3 | 261733552 | 261753218 | + | Odorant receptor | Or23 |  | 1,50E-04 | 12,33 | 3,62 |  | 1,54E-04 | 6,68 | 2,74 |  |  |
| AAEL019938 | 3 | 265359753 | 265360364 | - |  |  |  | 5,99E-04 | -8,52 | -3,09 |  | 1,50E-04 | -30,33 | -4,92 |  |  |
| AAEL003967 | 3 | 267744961 | 267747561 | + | calpain 4, 6, 7, invertebrate |  |  | 1,50E-04 | 5,28 | 2,40 |  | 1,50E-04 | 6,69 | 2,74 |  |  |
| AAEL020889 | 3 | 274040067 | 274252257 | + |  |  |  | 1,84E-04 | -3,42 | -1,77 |  | 1,50E-04 | -4,85 | -2,28 |  |  |
| AAEL019887 | 3 | 280597943 | 280922614 | - |  |  |  | 1,50E-04 | -4,88 | -2,29 |  | 1,50E-04 | -10,35 | -3,37 |  |  |
| AAEL014871 | 3 | 293945841 | 293991303 | - | methylenetetrahydrofolate dehydrogenase | |  | 1,52E-04 | 3,42 | 1,77 |  | 1,50E-04 | 3,98 | 1,99 |  |  |
| AAEL010752 | 3 | 293994666 | 294007242 | - |  |  |  | 1,50E-04 | 41,46 | 5,37 |  | 1,50E-04 | 30,14 | 4,91 |  |  |
| AAEL011068 | 3 | 294480222 | 294482635 | + |  |  |  | 1,57E-04 | -6,45 | -2,69 |  | 1,50E-04 | -16,93 | -4,08 |  |  |
| AAEL003619 | 3 | 301265516 | 301313082 | + | sodium/chloride dependent amino acid transporter | |  | 1,50E-04 | 5,95 | 2,57 |  | 1,50E-04 | 8,29 | 3,05 |  |  |
| AAEL006014 | 3 | 315325706 | 315431873 | - | heme peroxidase | HPX1 |  | 1,57E-04 | -3,46 | -1,79 |  | 1,50E-04 | -4,94 | -2,31 |  |  |
| AAEL009451 | 3 | 321271085 | 321274464 | + | actin |  |  | 1,50E-04 | -68,65 | -6,10 |  | 1,51E-04 | -14,22 | -3,83 |  |  |
| AAEL008757 | 3 | 326023639 | 326025510 | - |  |  |  | 3,19E-04 | 3,31 | 1,73 |  | 1,81E-04 | 3,90 | 1,96 |  |  |
| AAEL008752 | 3 | 326182117 | 326231983 | + |  |  |  | 2,63E-04 | -6,35 | -2,67 |  | 1,82E-04 | -7,59 | -2,92 |  |  |
| AAEL008753 | 3 | 326959568 | 326960678 | + |  |  |  | 1,50E-04 | 8,01 | 3,00 |  | 1,50E-04 | 5,26 | 2,40 |  |  |
| AAEL003299 | 3 | 330245556 | 330246486 | - |  |  |  | 1,50E-04 | 4,63 | 2,21 |  | 1,50E-04 | 5,54 | 2,47 |  |  |
| AAEL000878 | 3 | 332483976 | 332485212 | + | cuticle protein, putative |  |  | 1,50E-04 | -12,25 | -3,61 |  | 1,50E-04 | -19,38 | -4,28 |  | cuticle change |
| AAEL029005 | 3 | 332502995 | 332516375 | - |  |  |  | 1,66E-04 | -3,71 | -1,89 |  | 1,50E-04 | -6,73 | -2,75 |  |  |
| AAEL012856 | 3 | 339240504 | 339241235 | + |  |  |  | 1,50E-04 | 4,15 | 2,05 |  | 1,50E-04 | 4,56 | 2,19 |  |  |
| AAEL003294 | 3 | 340355818 | 340358440 | + | fibrinogen and fibronectin |  |  | 1,50E-04 | 6,03 | 2,59 |  | 1,50E-04 | 6,78 | 2,76 |  |  |
| AAEL023384 | 3 | 346885841 | 347093560 | + |  |  |  | 1,52E-04 | -3,86 | -1,95 |  | 1,50E-04 | -5,02 | -2,33 |  |  |
| AAEL003430 | 3 | 349299551 | 349304597 | + |  |  |  | 2,57E-04 | -3,83 | -1,94 |  | 1,50E-04 | -7,38 | -2,88 |  |  |
| AAEL003425 | 3 | 350173617 | 350191549 | - |  |  |  | 1,50E-04 | -3,13 | -1,65 |  | 1,50E-04 | -5,97 | -2,58 |  |  |
| AAEL003419 | 3 | 350343449 | 350395329 | - |  |  |  | 1,50E-04 | -3,68 | -1,88 |  | 1,50E-04 | -6,52 | -2,70 |  |  |
| AAEL011915 | 3 | 350886815 | 350898203 | - | F-box/lrr protein fragment |  |  | 1,50E-04 | -5,50 | -2,46 |  | 1,50E-04 | -7,31 | -2,87 |  |  |
| AAEL022536 | 3 | 350898569 | 350912778 | - |  |  |  | 1,50E-04 | -4,47 | -2,16 |  | 1,50E-04 | -4,39 | -2,13 |  |  |
| AAEL011923 | 3 | 350912882 | 350915210 | + | F-box/lrr protein fragment |  |  | 1,50E-04 | -3,33 | -1,73 |  | 1,50E-04 | -4,26 | -2,09 |  |  |
| AAEL025532 | 3 | 353273331 | 353288981 | - |  |  |  | 1,50E-04 | -3,58 | -1,84 |  | 1,50E-04 | -4,14 | -2,05 |  |  |
| AAEL008936 | 3 | 356273592 | 356276112 | + |  |  |  | 1,50E-04 | 3,60 | 1,85 |  | 1,50E-04 | 4,70 | 2,23 |  |  |
| AAEL008050 | 3 | 359610096 | 359611575 | - |  |  |  | 1,50E-04 | 11,02 | 3,46 |  | 1,50E-04 | 16,86 | 4,08 |  |  |
| AAEL000797 | 3 | 363906816 | 363908514 | - | dimethylaniline monooxygenase |  |  | 1,82E-04 | -3,36 | -1,75 |  | 1,50E-04 | -7,56 | -2,92 |  |  |
| AAEL011409 | 3 | 365206147 | 365207635 | + | Odorant receptor | GPROR100 | | 1,67E-04 | 4,43 | 2,15 |  | 1,50E-04 | 6,55 | 2,71 |  |  |
| AAEL023353 | 3 | 365480553 | 365481370 | + |  |  |  | 1,50E-04 | 4,93 | 2,30 |  | 1,50E-04 | 4,15 | 2,05 |  |  |
| AAEL011404 | 3 | 365525965 | 365526637 | - | C-Type Lectin (CTL19) | CTL19 |  | 2,02E-04 | 3,50 | 1,81 |  | 1,50E-04 | 5,17 | 2,37 |  |  |
| AAEL012353 | 3 | 365606138 | 365606779 | - | C-Type Lectin (CTL). | CTL15 |  | 1,50E-04 | 5,56 | 2,48 |  | 1,50E-04 | 10,74 | 3,42 |  |  |
| AAEL000678 | 3 | 395273413 | 395275588 | + | alpha-amylase |  |  | 1,55E-04 | -6,82 | -2,77 |  | 1,66E-04 | -5,91 | -2,56 |  |  |
| AAEL000668 | 3 | 395376851 | 395378817 | - |  | LOC5565448 | | 1,50E-04 | 7,07 | 2,82 |  | 1,50E-04 | 4,08 | 2,03 |  |  |
| AAEL004118 | 3 | 401730331 | 401731540 | - | aldo-keto reductase |  |  | 1,50E-04 | -3,27 | -1,71 |  | 1,50E-04 | -3,10 | -1,63 |  |  |
| AAEL018685 | MT | 10323 | 11457 | + | cytochrome b | CYTB |  | 1,50E-04 | 23,72 | 4,57 |  | 1,50E-04 | 37,14 | 5,21 |  |  |
| AAEL020853 | Unkn | 9152554 | 9153702 | + |  |  |  | 5,83E-04 | 4,31 | 2,11 |  | 1,50E-04 | 9,93 | 3,31 |  |  |
| AAEL008404 | Unkn | 66785895 | 66787465 | - | Clip-Domain Serine Protease family A. | CLIPA16 |  | 1,50E-04 | 4,50 | 2,17 |  | 1,50E-04 | 4,62 | 2,21 |  |  |
| AAEL022665 | Unkn | 79283960 | 79285298 | - |  |  |  | 2,31E-04 | -8,11 | -3,02 |  | 1,65E-04 | -10,62 | -3,41 |  |  |
