# Supplementary material for: A genomic amplification affecting a carboxylesterase gene cluster confers organophosphate resistance in the mosquito Aedes aegypti: From genomic characterization to high‐throughput field detection
Source: Evol Appl. 2021 Feb 16;14(4):1009–22. doi: 10.1111/eva.13177 (PMC8061265; doi:10.1111/eva.13177)

Detecting carboxylesterase AAEL023844 genomic amplification using multiplex TaqMan assay *(Cattel et al. 2020)*

- Primers and probes (used HPLC purified primers):

Gene	Tm (°C)	Forward (5'-3')	Reverse (5'-3')	Product length (bp)
CYP4D39	60	AGTCCTGGAAGTTCTGCACG	AAGGCGACTTTCCGACGAAT	132
AAEL023844	60	TATAGCAGGAAGCGGCGATG	AATCCCAAGGGACCCAATCG	102
CYP4D39**	60	[HEX]AAGGAGGCAAACCCCGATAA[BHQ1]		
AAEL023844**	60	[FAM]TATAGTGCAGGAGGGGGTCA[BHQ1]		

- qPCR mix (per well):

Reaction mixture	Volume (µL)
qPCR Master Mix (Bio-Rad)	12.5
CYP4D39_up (10µM)	2.25
CYP4D39_down (10µM)	2.25
CYP4D39_probes (HEX) (10µM)	0.625
AAEL023844_up (10µM)	2.25
AAEL023844_down (10µM)	2.25
AAEL023844 probes (FAM) (10µM)	0.625
Nuclease free water	1.25
DNA sample (0,5 ng/µL)	1
Total volume	25

- qPCR program:

1) 95°C for 10:00
2) 95°C for 0:10
3) 60°C for 0:45<br>+ Plate Read (FAM/HEX)
4) GOTO 2), 39 more times<br>END

- Data interpretation (amplification curve):

The figure below show typical qPCR profiles obtained for both the CCE gene AAEL023844 and the control gene from a positive sample (presence of CCE gene amplification) and a negative sample (absence of CCE amplification). CNV in the positive sample as compared to the negative sample can be estimated using the ddCt relative quantification method.

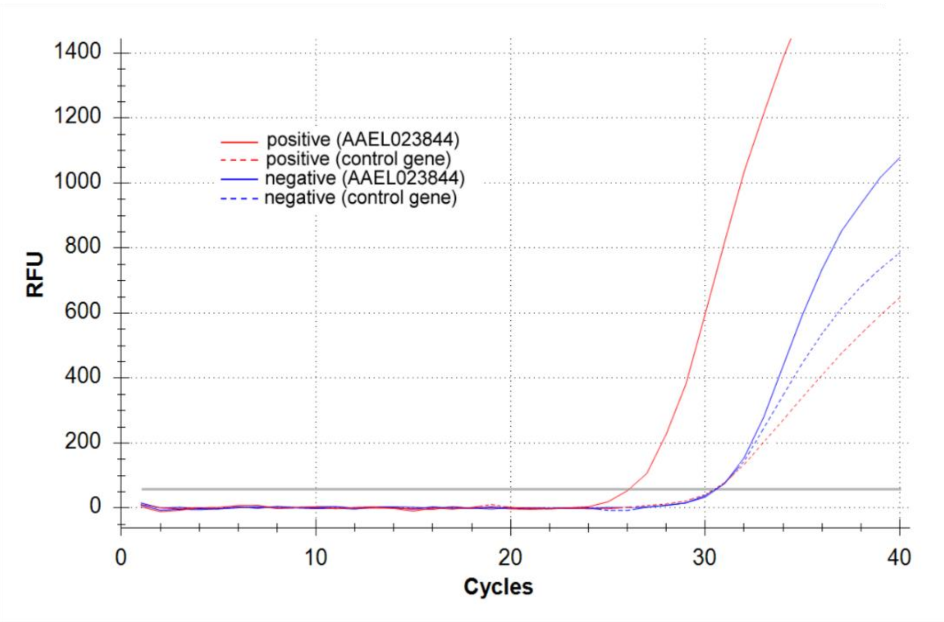

Supplement: Supplementary file 5 — File S1 [file EVA-14-1009-s001.pdf]
